# Febrile Seizure in Children Attending a Tertiary Care Centre in Western Nepal: A Descriptive Cross-sectional Study

**DOI:** 10.31729/jnma.6322

**Published:** 2021-04-30

**Authors:** Bandana Shrestha, Arjun Bhattarai, Nabraj Subedi, Nirmala Shrestha

**Affiliations:** 1 Department of Pediatrics, Gandaki Medical College and Teaching Hospital, Pokhara, Nepal; 2 Department of Community Medicine, Gandaki Medical College and Teaching Hospital, Pokhara, Nepal

**Keywords:** *febrile seizure*, *prevalence*, *tertiary centre*

## Abstract

**Introduction:**

Understanding a child with any episode of febrile seizure is important so that special attention could be given. The objective of this study was to find the prevalence of febrile seizure in children attending a tertiary centre in western Nepal.

**Methods:**

A descriptive cross-sectional study was performed in a tertiary care centre at the department of Pediatrics after taking approval from the Institutional Review Committee. Study was conducted among the children presented with febrile seizure from 18th October 2017 to 12th April 2020. Patient files were retrospectively reviewed. Convenience sampling method was used. Data and descriptive analysis were done using Statistical Package for the Social Sciences version 25. Point estimate at 95% Confidence Interval was calculated along with frequency and percentage for binary data.

**Results:**

Of the total 4701 admitted children during a study period, 217 (4.61%) (3.41-5.81 at 95% Confidence Interval) children had febrile seizure. Out of them, 154 (70.9%) male and 63 (29.1%) female with 168 (77.4%) simple and 49 (22.6%) complex febrile seizure. The mean age of presentation was at 23.2±13.61 months whereas mean age for male and female were 22.99±13.86 months and 23.73±13.09 months respectively. Recurrent febrile seizure noted in 68 (31.3%) children and fever in half the cases 110 (50.7%) was caused by Upper Respiratory Tract Infection.

**Conclusions:**

Simple febrile seizure was more common and the peak age of presentation was in the second year of life and more commonly in male. One third of febrile seizures were recurrent and half the children had upper respiratory tract infection as the most common etiology of fever.

## INTRODUCTION

Febrile seizure is seen 2-5% of the children aged 6months to 5 years and are triggered by fever.^1^ The International League Against Epilepsy (ILAE) has defined as seizure events in children, featured with a temperature over 38°C without any evidence of acute electrolyte imbalances or central nervous system infection.^2^

Although seizure in children is a significant cause of morbidity and mortality, febrile seizure is still considered as benign and self-limiting. But it is emotionally perceived as terrifying and anxiety provoking as witnessed by the parents.^1,3^ An understanding of febrile seizure is an issue of interest to reassure the parents and provide appropriate management. Children of 2%-5%, experienced febrile seizure in Western Europe and United States.^1^ However, data related to febrile seizure in our country is limited.

This study aims to find out the prevalence of febrile seizure in children admitted in a tertiary centre in western Nepal.

## METHODS

This is a descriptive, cross sectional study, carried out in Gandaki Medical College and Teaching Hospital, in Kaski district of Western Nepal. The study was conducted among the children, admitted from 18th October 2017 to 12^th^ April 2020, consistent with diagnosis of febrile seizure. An ethical approval was obtained from the Institutional Review Committee of Gandaki Medical College prior to the study with the reference number (065.2077.2078). Records of all the children aged 6 months to 6 years, admitted with a diagnosis of febrile seizure were included in the study. Those who had prior history of afebrile seizures, children already on regular anticonvulsants treatment and those who had central nervous system abnormalities on radioimaging and those with neurologically abnormal child were excluded from the study. In addition, patient with grossly inadequate data were also excluded from the study. The convenient sampling method was applied and the sample size was calculated as

$$\begin{array}{ll} \text{n} & ={\text{Z}}^{\text{2}}\times \text{p}\times \text{q}/{\text{e}}^{\text{2}}\\ & ={\left(2.576\right)}^{2}\times .084\times .916/0.015^{\text{2}}\\ & =2270 \end{array}$$

where,

n = required sample size
Z = 1.645 at 90% Confidence Interval
p = prevalence of febrile seizure in population, on average 16 %^4^
q = 100-p
e = margin of error, 1%

Taking double the sample size was calculated to be 4540. Taking a 10% non-response rate, the sample size became 4001. However, the total sample size of 4701 was taken. Children with febrile seizure were identified from pediatric ward admission register and their record files were obtained from medical record section and detail required data was retrieved.

Information collected based on the patients' demographic and clinical data regarding type of seizure, duration, number of episodes, interval from onset of fever to seizure, past episodes of febrile seizure, family history of febrile seizure and epilepsy, cause of fever and hemoglobin level at the onset of seizure. Febrile seizure was labelled as simple, complex and recurrent. Simple is a short generalized seizure, of duration of less than 15 minutes, not recurring within 24 hours, occurring during a febrile episode whereas complex is a focal, or generalized and prolonged seizure, of a duration of greater than 15 minutes, recurring more than once in 24 hours, and/or associated with postictal neurological abnormalities.^1^ Those children who had a past history of at least one febrile seizure and admitted with another episode of febrile seizure was regarded as recurrent febrile seizure. Children considered anemic when his/her hemoglobin recorded below 11mg/dl.^5^

Data entered using Microsoft Excel and analyzed using Statistical Package Social Sciences version 25. Descriptive statistical tools like frequency, percentage, mean, standard deviation and tables were used to express the result. Point estimate at 90% confidence interval was calculated along with frequency and percentage for binary data.

## RESULTS

A total 4701 children were admitted during the study period. Among them, admitted children with febrile seizure were 217 (4.61%) and were enrolled in the study. Out of them, 154 (71%) were male and 63 (29%) were female children with the proportion male to female 2.4:1. The mean age of presentation was at 23.2±13.61 months whereas mean age for male and female were 22.99±13.86 and 23.73±13.09 months respectively. The peak age was in the second year of life with more than half of the children presented by 13 to 24 months of age and then the prevalence decreases as age of the child increases (Table 1).

**Table 1:** Age wise distribution across male and female gender (n =217).

Age Groups (in months)	Gender		Total
	Male n (%)	Female n (%)	n (%)
≥6	38 (17.5)	9 (4.2)	47 (21.7)
13 to 24	77 (35.5)	37 (17)	114 (52.5)
25 to 36	18 (8.3)	12 (5.5)	30 (13.8)
37 to 48	12 (5.5)	0 (0)	12 (5.5)
≥49	9 (4.2)	5 (2.3)	14 (6.5)
Total	154 (71)	63 (29)	217 (100)

The mean age at the onset of first febrile seizure episode was 19.41±11.12 months. Simple febrile seizures were seen in 168 (77.4%) children and complex seizures in 49 (22.6%) cases. Of them, recurrent febrile seizure noted in 68 (31.3%) children while remaining 149 (68.7%) presented with first episode of febrile seizure (Table 2).

**Table 2:** Clinical Characteristics of febrile seizure in children (n= 217).

Variables	Categories	Febrile Seizure		Total
		First episode	Recurrent episode	
		n (%)	n (%)	n (%)
Sex	Male	100 (46.1)	54 (24.9)	154 (71)
	Female	49 (22.6)	14 (6.4)	63 (29)
Types of seizure	Typical	116 (53.4)	52 (24)	168 (77.4)
	Atypical	33 (15.2)	16 (7.3)	49 (22.6)
Family history of febrile seizure	Yes	8 (3.7)	10 (4.6)	18 (8.3)
	No	141(65)	58 (26.7)	199 (91.7)
Family history of Epilepsy	Present	2 (0.9)	4 (1.8)	6 (2.7)
	Absent	147 (67.8)	64 (29.5)	211 (97.3)
Age at first episode of febrile seizure	>12 months	111 (51.1)	39 (18)	150 (69.1)
	≤12 month	38 (17.5)	29 (13.4)	67 (30.9)
Temperature at the time of seizure (degree celsius)	37.8-40	131(60.4)	58 (26.7)	189 (87.1)
	>40	18 (8.3)	10 (4.6)	28 (12.9)
Time duration between onset of fever and initial seizure	Within one hour	25 (11.5)	26 (12)	51 (23.5)
	> one hour	124 (57.1)	42 (19.3)	166 (76.4)
Seizure frequency in single febrile illness	Single	109 (50.2)	49 (25.6)	158 (72.8)
	More than one	40 (18.4)	19 (8.7)	59 (27.2)
Hb %	Anemia	81 (37.3)	33 (15.2)	114 (52.5)
	No Anemia	68 (31.3)	35 (16.2)	103 (47.5)

Among the febrile seizure in recurrence, 46 (21.1%) children presented with first recurrence, 13 (6%) presented with second recurrence, five presented with third recurrence and remaining four presented with fourth or more seizure recurrence (Table 3).

**Table 3:** Distribution of recurrent febrile seizure by number of episodes (n= 68).

Episodes of recurrent febrile Seizure	n (%)
First recurrence	46 (21.2)
Second recurrence	13 (6.0)
Third recurrence	5 (2.3)
Fourth or more recurrence	4 (1.9)

Most of the children 158 (72.8%) had single episode of seizure during single febrile illness, whereas 40 children 40 (18.4%) had two-episode,^13^ 13 (6%) had three episodes and one child had four episodes of seizure during a single febrile episode.

Eighteen (8.3 %) children had family history of febrile seizure whereas a history of epilepsy in the family was found in 5 (2.3%) (Table 2). The mean body temperature upon admission was 38.24±.05 degrees centigrade with the maximum recorded was 41.11 and minimum was 37.22 degrees centigrade. Upper respiratory tract infection was the most common cause of fever in children (50.7%) followed by lower respiratory tract infection (19.8 %) and urinary tract infection (12.4%) (Table 4).

**Table 4:** Etiology that triggered fever among the children with febrile seizure (n= 217).

Etiology	n (%)
Upper Respiratory Tract Infection	110 (50.6)
Lower Respiratory Tract Infection	40 (18.4)
Urinary Tract Infection	27 (12.4)
Acute Gastroenteritis	13 (6.0)
Acute Serous Otitis Media	3(1.4)
Enteric Fever	3 (1.4)
Mumps	2 (0.9)
Sepsis	2 (0.9)
Nonspecific	17 (7.8)

## DISCUSSION

Febrile seizure is the most common cause of seizure and children may develop seizure at any point during a febrile disease and may only develop a fever after their seizure.^1^ The present study found that febrile seizure was seen in 4.6% of the admitted children, which is comparable to the other studies conducted in rural Tanzania and Korean children which noted febrile seizure to be 4% and 6.9% respectively.^6,7^ In contrast, Delpisheh A, et al found that the prevalence of febrile seizures in Iran was 47%.^8^ The high prevalence in their study is due to the fact that their study noted prevalence of febrile seizure in all the seizure patients whereas present study noted febrile seizure among all the total admitted patients. Our study found the peak age was in the second year of life with almost more than half presented by 13 to 24 months of age. This is similar to the other studies as well.^6,8,9^ However, the study revealed that the prevalence gradually decreases as theage of the child increases. This finding is consistent with the study done by Delpisheh A, et al.^8^ The mean age of presentation was at 23.2±13.6 months of age, which is similar to the results of other studies.^9,10,11^ The male predominance was noted in our study which is similar to the findings of the other previous studies.^4,9,10,12-14^ This could be because of the fact that it was an institutional based study where gender bias for health seeking behavior could be the reason.

Simple febrile seizure ranging from 70 to 84%, is more commonthan complex febrile seizure which ranged from 11% to 16% in different studies across the world.^8,11,15^ A study from Nepal also showed simple febrile seizure is common than complex febrile seizure and it was comparable to our study.^9,13^ However this finding is contrary to the another study conducted in rural Tanzania where the prevalence upto 70% complex seizure were documented.^6^

Generalized tonic clonic seizure was the most common presentation in our study. Febrile status epilepticus noted in 2.3% of the cases, which was less than 4.5% and 9% reported in the previous studies.^4,6^ The reduced prevalence of febrile status epilepticus in our study may partly the result of an improved health education and on timely arrival to the hospital for health access.

Only 8.3% of the cases were found to have positive family history of febrile seizure in our study which is comparable to 8% conducted by Mwipopo EE, et al. in China.^16^ In contrast to our findings, other previous studies showed that family history of febrile seizure varies from 20% to 50% of the cases, which was higher than this study.^14,15,17^ The difference could be because of the fact that parents may not be aware of their past illness and not able to recall as they were too young to remember the past medical illness. Besides all these, probably due to genetic potential in different populations could be the reason.

In the present study, the most common etiology for febrile seizure was upper respiratory tract infection (50.7%). Many previous studies also reported URTI as the predominant cause.^9,13,14,17^ The cause of URTI in our study presumed to be viral origin, based on their clinical characterizations and its course of illness as we did not have laboratory facility to isolate and identify virus. Following URTI, other causes are lower respiratory tract infection (19.2%), UTI (12.4%), nonspecific febrile illness and gastrointestinal infection. The secondary cause of febrile seizure in our study and other previous studies concluded the same findings.^6,14^

This study found 31.3% of children had recurrent febrile seizure, and it was comparable to the other studies which showed that the recurrence of febrile seizure was 33%, 32.5%.^9,17^ However, the likelihood of recurrence in the present study is higher than few previous studies which noted that prevalence of recurrent seizure were 20.5%, 19.92%.^11,15^ The difference could be due to the fact that environmental factors predisposes the children to have a fever that subsequently predispose to seizure among the genetically potential populations. Previous literature revealed that iron deficiency anemia, is a risk factor for febrile seizures.^12,18^ This study also revealed that more than half of the cases, i.e. 52.5% had nutritional anemia associated with iron deficiency.

However, although our investigations have reached its aim, there did exist some limitation. It is a single institutional based finding with limited sample size. Despite with all the efforts to records data, information could not be reviewed in every aspect as the data is collected retrospectively.

## CONCLUSIONS

Prevalence of febrile seizure was comparable to studies from rural Tanzania and Korean children. Simple febrile seizure being encountered more, notably more common in malesand the peak age of presentation wasin the second year of life. Recurrent febrile seizure noted in one third of the cases. However positive family history of febrile seizure was low as compared to other previous studies. This research may aid to estimate frequency and rates of recurrence in our population which may avoid unnecessary diagnostic and therapeutic interventions.
